# Phytochemical and nanoparticle-based therapeutic potential of *Sphaeranthus indicus* against hepatocellular carcinoma via cryptomeridiol targeting

**DOI:** 10.3389/fonc.2025.1691905

**Published:** 2025-11-14

**Authors:** Ritika Ravindra Konduskar, Abhinandan R. Patil, Somnath D. Bhinge, Gourav Rameshlal Chawla, Yash Bhagavan Yadav

**Affiliations:** 1 Bharati Vidyapeeth College of Pharmacy, Kolhapur, Maharashtra, India; 2 Department of Pharmaceutics, D Y Patil Education Society Deemed To Be University, Kolhapur, Maharashtra, India; 3 Department of Pharmaceutical Chemistry, Krishna Institute of Pharmacy, Krishna Vishwa Vidyapeeth (Deemed to be University), Karad, Maharashtra, India; 4 Department of Pharmaceutics, Krishna Institute of Pharmacy, Krishna Vishwa Vidyapeeth (Deemed to be University), Karad, Maharashtra, India

**Keywords:** *Sphaeranthus indicus*, cryptomeridiol, silver nanoparticles (Ag NP), hepatocellular carcinoma (HCC), apoptosis

## Abstract

**Background:**

Cancer remains a major global health challenge, and despite advances in chemotherapy, there is a need for safer plant-derived therapeutics. *Sphaeranthus indicus* (Si) (East Indian globe thistle), traditionally used in herbal medicine, exhibits anticancer potential. This study evaluated the cytotoxic activity of Si alcoholic extract, its bioactive fraction, and biosynthesized silver nanoparticles (SiAgNPs) against hepatocellular carcinoma (HepG2) cells.

**Methods:**

Alcoholic extract of Si extract was fractionated, and GC–MS identified cryptomeridiol as the major bioactive compound. Structural confirmation was performed using IR, ^1H NMR, and UV–Vis spectroscopy. SiAgNPs were synthesized using Si extract and characterized by UV–Vis, FTIR, FESEM, and XRD. Cytotoxicity was assessed by CCK-8 assays, while apoptosis was confirmed morphologically. Molecular docking evaluated the binding of cryptomeridiol with hepatocellular carcinoma-associated protein targets (PDB IDs: 8QAL, 8QAN, 8QAP, 8QAR, 8QAZ).

**Results:**

IR spectra confirmed hydroxyl and olefinic functional groups in cryptomeridiol, while ^1H NMR showed characteristic methyl, methylene, hydroxyl methine, and olefinic proton signals. SiAgNP formation was indicated by a color change (yellow → brown) and a surface plasmon resonance peak at 437 nm. FTIR of SiAgNPs revealed reduced intensities of O–H, C=O, and C–O bands, confirming phytochemical involvement in nanoparticle stabilization. FESEM showed spherical nanoparticles with an average size of 38.35 ± 16.42 nm, and XRD analysis confirmed their crystalline FCC structure with a crystallite size of ~16.8 nm. Cytotoxicity assays demonstrated IC_50_ values of 44.93 μg/mL (Si extract), 43.87 μg/mL (cryptomeridiol), and 42.16 μg/mL (SiAgNPs), comparable to 5-fluorouracil (43.16 μg/mL). All treatments inhibited HepG2 proliferation by >75% and induced apoptosis-like morphological changes. Molecular docking revealed cryptomeridiol interacted strongly with all selected protein targets, with binding energies ranging from −7.1 to −8.1 kcal/mol, involving hydrogen bonds, alkyl, and van der Waals interactions.

**Conclusion:**

Si extract, cryptomeridiol, and SiAgNPs are well-characterized, biologically active agents that induce apoptosis and inhibit HepG2 proliferation. These findings highlight cryptomeridiol as a potent phytochemical scaffold and demonstrate the translational potential of combining phytochemicals with nanotechnology for hepatocellular carcinoma therapy through the *in-vitro model*.

## Introduction

Cancer is the world’s second largest cause of mortality, despite significant breakthroughs in medical research (1–3). Hepatocellular carcinoma (HCC) is the most frequent primary liver tumor and a leading cause of cancer-related mortality globally (4, 5). It ranks the fifth most prevalent primary liver malignancy and the second most significant cause of cancer-related mortality globally (6, 7). In 2020, approximately 905,700 new liver cancer cases were reported worldwide, with an age-standardized incidence rate (ASIR) of 9.5 per 100,000 individuals (8). Notably, 75% of cases occur in Asia, with China alone contributing over 50% (9). Males are disproportionately affected, with incidence rates two to three times higher than in females (9). Mortality remains high, with 830,200 deaths recorded in 2020 and an age-standardized mortality rate (ASMR) of 8.7 per 100,000 (8).

The major risk factors for the development of HCC are chronic alcohol intake, hepatitis B and C virus infections, and nonalcoholic fatty liver disease (10). Various therapies available for HCC patients include systemic chemotherapy, microwave, cryotherapy, and radiofrequency ablations, radiation therapy, and targeted treatments. Sorafenib, approved in 2007, inhibits receptor tyrosine kinases like VEGFR and PDGFR, prolonging survival in unresectable HCC (11). Regorafenib, approved in 2017, serves as a second-line treatment following sorafenib failure (11). Immunotherapy using nivolumab, a PD-1 inhibitor, enhances T-cell activation against cancer cells. Lenvatinib (approved in 2018) targets VEGFR and FGFR, while cabozantinib inhibits multiple oncogenic pathways (11).

Despite these advancements, resistance and tumor heterogeneity remain challenges. Emerging strategies, including combination therapies and novel drugs aim to optimize efficacy and overcome therapeutic resistance also. Moreover, chemotherapy is routinely used in HCC patients to manage disease development (12, 13). However, chemotherapeutic agents often cause severe adverse effects that cannot be completely prevented (14). Natural herbal medications offer several advantages over conventional chemical pharmaceuticals in mitigating the adverse effects of chemotherapy (13, 14).

In this context, *Sphaeranthus indicus* (Si) was selected for the study of HCC due to its promising anti-cancer potential. Previous research has identified several bioactive phytoconstituents from Si with significant cytotoxic potential. Notably, a study by Nahata et al. (2013) reported that β-sitosterol, isolated from the petroleum ether extract of Si, exhibited significant cytotoxic potential (15). Isolated β-sitosterol were effective against several human cancer cell lines, including HL-60 (leukemia), A549 (lung), HeLa (cervical), HEP-2 (Liver) and MCF-7 (breast) cell lines. Furthermore, the green synthesis of silver nanoparticles (AgNPs) using Si extract has yielded nanoparticles ranging from 16–20 nm in size, demonstrating enhanced biological and anticancer activities (16). The primary focus was on the induction of apoptosis through the mitochondrial-dependent pathway in HL-60 cells. Moreover, the US patent US20080199550A1 pertains to the therapeutic potential of Si and its extracts, particularly highlighting their anticancer properties (17). The patent discloses formulations containing extracts of Si and their use in inhibiting the growth of cancer cells. The patent claims that the active constituents, including β-sitosterol and 7-hydroxyfrullanolide, exhibit significant cytotoxic activity against various human cancer cell lines. The potential of Si extract as a natural source of anticancer agents underscores its promising therapeutic applications in oncology. However, the traditional approach of using crude plant extracts poses limitations, particularly regarding target-specific activity. In the current era of nanotechnology, the development of metallic nanoparticles emerges as a viable strategy to enhance the bioavailability and target specificity of phytoconstituents. By leveraging the unique properties of metallic nanoparticles, it is possible to improve the therapeutic efficacy of Si-derived compounds, thereby advancing their application in cancer treatment. Metal-based nanoparticles can be synthesized using a variety of physical and chemical techniques. The conventional methods involve laborious procedures such as chemical reduction, oxidation, hydrolysis, calcination, and condensation to prepare various nanoparticles (18). The advancement of green chemistry and biological preparation techniques has made it possible to create an environmentally friendly process for Np preparation. Plant extracts and a wide variety of microorganisms can be utilized to create nanoparticles from over the last few years (19). Silver nanoparticles (AgNPs) are nobel metal nanoparticles that are gaining attention in nanotechnology research due to their unique qualities such as better optoelectronic capabilities, large surface area, and biocompability (20). AgNPs are recommended in anticancer medicines because they are more biocompatible than traditional therapies. Thus, they may be used for site-specific delivery and drug encapsulation to boost medication efficacy and avoid the unwanted harmful effects of large-sized particles (21, 22).

**Figure 1:**
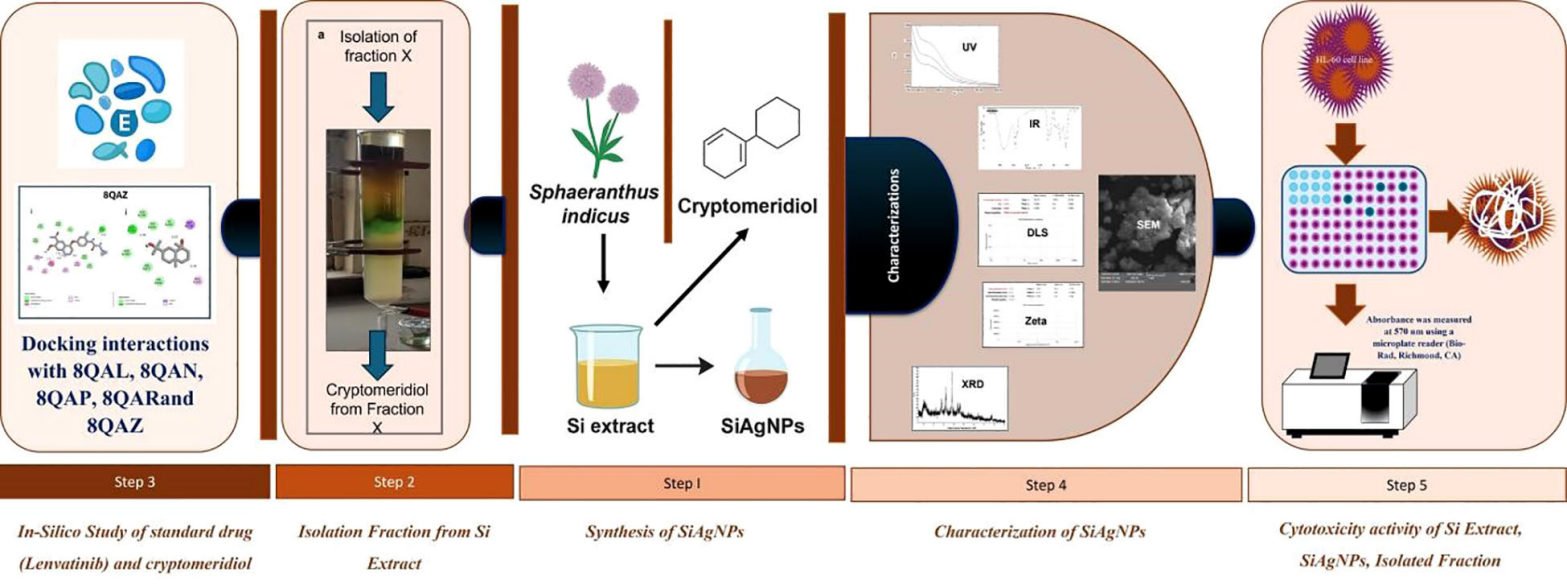
Detailed representation of the research work.

Despite extensive studies on the pharmacological properties of Si, limited research has focused on its molecular mechanisms and nanotechnology-based applications for HCC. Furthermore, there is a lack of comprehensive studies integrating phytochemical analysis, nanoparticle synthesis, and molecular docking to elucidate the mechanistic interaction between Si-derived molecules and HCC-associated protein targets. This study addresses these gaps by isolating cryptomeridiol, synthesizing Si-mediated silver nanoparticles (SiAgNPs), and evaluating their cytotoxic and apoptotic potential against HepG2 cells. Therefore, the current investigation aims to identify the potential cytotoxicity of ethanol extracts and their derived fractions of Si extract on HCC using HepG2 cells. (SiAgNPs) Although Si is an effective medicinal plant, its cytotoxicity against lung, ovary, prostate, neuroblastoma, breast, and colon cancers and the responsiveness of cell lines to the plant extract have not been studied (**Figure 1**). The current study aims to determine the cytotoxic and apoptotic potential of the alcoholic extract and isolated components of Si on HCC (HepG2).

## Materials and methods

### Chemicals and reagents

Fetal bovine serum (FBS), dimethylsulfoxide (DMSO), trypsin-ethylenediaminetetraacetic acid (EDTA), 3-(4,5-dimethylthiazol-2-yl)-2,5-diphenyltetrazolium bromide (MTT), antimycotic, and Dulbecco’s minimum essential low glucose medium (DMEM) were acquired from GIBCO BRL (Gaithersburg, MD). In order to assist M/s. Sigma Chemical (India) and Silymarin (SIL) were bought. All additional chemicals were of an analytical grade.

### Plant material, extraction and isolation

The whole plant of Si was collected in the month of November 2023 from the Western Ghats region of Maharashtra, India, primarily spanning the Sahyadri mountain range, located at latitude 15.6° N to 20.0° N and longitude 73.2° E to 74.5° E.A voucher specimen (RRK-01) has been stored in the Botany Department’s herbarium at Shivaji University in Kolhapur, MS, India. Plants were thoroughly cleansed and the plants were shade-dried to a consistent weight for 15–20 days at (room temperature22-26°C.). Dried plants were broken into small bits to ensure consistent powder size, homogenized, and passed through a 60-mesh sieve (23). The plant powder was subjected to Soxhlet extraction using ethanol as the solvent. The resulting extract was dried under a nitrogen (N_2_) gas stream, and the yield percentage was calculated (23). A Brucker DNP-NMR was used to get the NMR spectra (400 and 500 MHz).

### Isolation procedure

The extract was suspended in distilled water and sequentially partitioned with appropriate solvents. The alcoholic fraction was subjected to silica gel column chromatography and eluted using a mobile phase of chloroform and benzene in a 7:3 ratio. Gas chromatography-mass spectrometry (GC-MS) was used with an SH-Rxi-5Sil MS column (30 m 0.25 mm 0.25 m, Shimadzu, Japan). The carrier gas was helium (99.999%), and the GC-MS apparatus received 1 µL of Fraction X solution at a flow rate of 1.01 mL/min. The oven temperature was set to 50°C at first, then steadily increased to 150°C at a rate of 2°C per minute, with a final hold at 250°C for 3 minutes, as described earlier. The temperatures of the interface and the ion source were set to 270°C and 200°C, respectively. The retention time (RT) and MS data were confirmed by comparing them to reference data from the SAIF-DST CFC at Shivaji University, Kolhapur, Maharashtra 416004, obtained through a NIST library search. Finally, the peak area under the curve was calculated to establish the components percentage composition. The JASCO V 630 UV-Vis spectrophotometer was used to record the cryptomeridiol’s ultraviolet (UV) visible (Vis) spectra, and FTIR spectroscopy (JASCO FTIR 4600, which covers the 4000–600 cm^−1^ range) was used to get the Fourier-transform infrared (FTIR) spectra.

### Synthesis of Si plant extract silver nanoparticles

Ten grams of Si plant extract was dissolved in 100 mL of boiling double distilled water for 10 minutes. Following that, the plant extract was filtered using Whatman filter paper no. 1 and utilized for further processing (24). The reaction was carried out in a 250 mL conical flask containing 100 mL of 1 mM AgNO_3_ solution and 10 mL of Si plant extract and swirled well for 30 minutes. After 30 minutes, the faint brown solution became intense brown, which indicates the formation of SiAgNPs.

### Characterization of SiAgNPs

The UV-visible spectra of the synthesized SiAgNPs were recorded using a Shimadzu UV-1900 spectrophotometer (Japan), covering a wavelength range of 200–800 nm with a resolution of 1 nm. To confirm the functional groups, present in the SiAgNPs, Fourier Transform Infrared (FTIR) spectroscopy was performed using a Bruker Alpha II spectrometer (Germany), with a scanning range from 400 to 4000 cm^−1^. The crystallographic structure of the SiAgNPs was analyzed by X-ray diffraction (XRD) using Cu Kα radiation (λ = 1.54060 Å) and a nickel monochromator. The diffraction patterns were obtained in the 2θ range from 10° to 80° using an X’pert PRO PAN analytical diffractometer. The hydrodynamic diameter and polydispersity index (PDI) of the Si*–*mediated silver nanoparticles (AgNPs) were determined using a Malvern Zetasizer (Dynamic Light Scattering, DLS) system. Deionized water was used as the dispersant, with a refractive index (RI) of 1.330 and viscosity of 0.8872 mPa·s. The measurements were performed at a system temperature of 25.069°C. The nanoparticle suspension was suitably diluted with the dispersant to obtain an optimal count rate of 27.5 kcps, and measurements were taken at a measurement position of 4.65 mm with attenuator setting 11. Each run was recorded for a duration of 50 s, and the average of three consecutive readings was reported to ensure data reliability. The surface charge and colloidal stability of the Si–mediated silver nanoparticles (AgNPs) were evaluated using a Malvern Zetasizer (Zeta Potential Analyzer) based on Laser Doppler Electrophoresis (LDE). Deionized water was used as the dispersant with a refractive index (RI) of 1.330, viscosity of 0.8872 mPa·s, and dielectric constant of 78.5. The measurements were performed at a system temperature of 25.0°C. The nanoparticle suspension was appropriately diluted to achieve a count rate of 409.3 kcps, and zeta potential was determined using 13 consecutive runs to ensure statistical accuracy. The measurement was carried out at a position of 2.005 mm with an attenuator setting of 9. Additionally, the morphology and surface features of the SiAgNPs were examined through Field Emission Scanning Electron Microscopy (FESEM) using a MIRAJ 3 instrument (TESCAN Ltd., Czech Republic). Prior to imaging, the nanoparticle samples were drop-cast onto a clean silicon wafer and allowed to air-dry. The samples were then sputter-coated with a thin layer (~5 nm) of gold to prevent charging. FESEM imaging was performed with a UHR pole piece electron microscope operating at an accelerating voltage of 200 kV. Images were captured at multiple magnifications to analyze particle size, shape, and surface characteristics, and at least five fields per sample were examined to ensure reproducibility.

### *In vitro* assay for cytotoxic activity (MTT assay)

HCC HepG2 cells were cultured in RPMI-1640 medium (Gibco), supplemented with 10% heat-inactivated fetal bovine serum (FBS, Gibco, Invitrogen; Cat. No. 10270106) and 1% Antibiotic-Antimycotic 100X solution (Thermo Fisher Scientific; Cat. No. 15240062). The cells were incubated at 37°C in a humidified incubator with 5% CO_2_. The cytotoxicity of the water-soluble AME was assessed using the MTT assay. Normal and cancer cells (1 × 10³ cells per well) were seeded into 96-well plates containing 100 µL of medium per well (Costar Corning, Rochester, NY). Following overnight incubation, water-soluble SiAgNPs was added at different concentrations (100, 200, 300, 400, and 500 µg mL^-1^) to five replicate wells. After treatment with water-soluble SiAgNPs for 1, 2, 3, 4, and 5 days, 20 µL of MTT solution (5 mg mL^-1^, pH 4.7) was added to each well, followed by incubation for an additional 4 hours. The supernatant was carefully removed, and 100 µL of DMSO was added to each well. The plates were agitated for 15 minutes to dissolve the formazan crystals.

Absorbance was measured at 570 nm using a microplate reader (Bio-Rad, Richmond, CA), with blank wells serving as controls. Three independent experiments were performed. The effect of water-soluble AME on human normal liver and HCC cells was expressed as a percentage of cell viability, calculated using the following formula (Equation 1):

(1) $$\text{\% cell viability}=\frac{Absorbance\;of\;treated\;cell}{Absorbance\;of\;control\;cell}\;\times \;100$$

### Cell viability analysis

Cell viability was measured using the CCK-8 test (25). The cells were plated in 96-well culture plates at 105 cells/mL density and adhered at 37 °C for 12 hours. The cells were then subjected to varying Si alcoholic extract, isolated fraction and SiAgNPs. After exposure, the medium with different Si alcoholic extract, isolated fraction and SiAgNPs concentration was discarded, and the cells were rinsed once with PBS (26). Fresh medium containing CCK-8 reagent were added to each well. After an hour at 37 °C, the absorbance of OD450 was measured using an ELISA plate reader (Tecan, Switzerland). An inverted microscope was used to study morphological changes following exposure to Si alcoholic extract, isolated fraction and SiAgNPs.

### Statistical analysis

All *in vitro* studies were performed in triplicate, with at least three independent replications. Data are presented as means ± standard deviations. Differences between control, standard Si extract fraction, and AgNP-treated cells were analyzed using Student’s t-test. For comparisons involving multiple groups, one-way ANOVA followed by appropriate *post-hoc* tests was performed. Confidence intervals were calculated, and P-values< 0.05 were considered statistically significant.

### Molecular docking

Autodock version 4.2 was initially used to conduct ligand-receptor interactions. To target specific receptors, lenvatinib and cryptomeridiol were prepared and optimized as crystallized ligands (27, 28). The Autodock 4.2 software was used to determine the free energy of the ligand-receptor complexes (29). The following identifiers were used to acquire protein structures from the RCSB Protein Data Bank (PDB): 8QAL (the first bromodomain of BRD4 in complex with acetyl-pyrrole derivative compound 83), 8QAN (the first bromodomain of BRD4 in complex with acetyl-pyrrole derivative compound 79) 8QAP (the first bromodomain of BRD4 in complex with acetyl-pyrrole derivative compound 2), 8QAR (the first bromodomain of BRD4 in complex with acetyl-pyrrole derivative compound 98), 8QAZ (BRPF1 bromodomain in complex with acetyl-pyrrole derivative compound 3). The ACDChemSketch tool was used to create 3D structures for Lenvatinib and cryptomeridiol. To discover probable binding sites, Autodock 4.2 was used, along with local docking and blind docking approaches. The binding pocket was identified by creating a grid box that encompassed the whole surface of the protein receptors. Discovery Studio Visualiser and PyMOL 1.7.4 were used to analyze residue interactions, which included van der Waals and hydrogen bonds. The Q-SiteFinder service was used to anticipate the amino acids found in the binding sites of various ligand-receptor complexes.

## Results

### Extraction, isolation of isolates from Si extract

**Figure 2:**
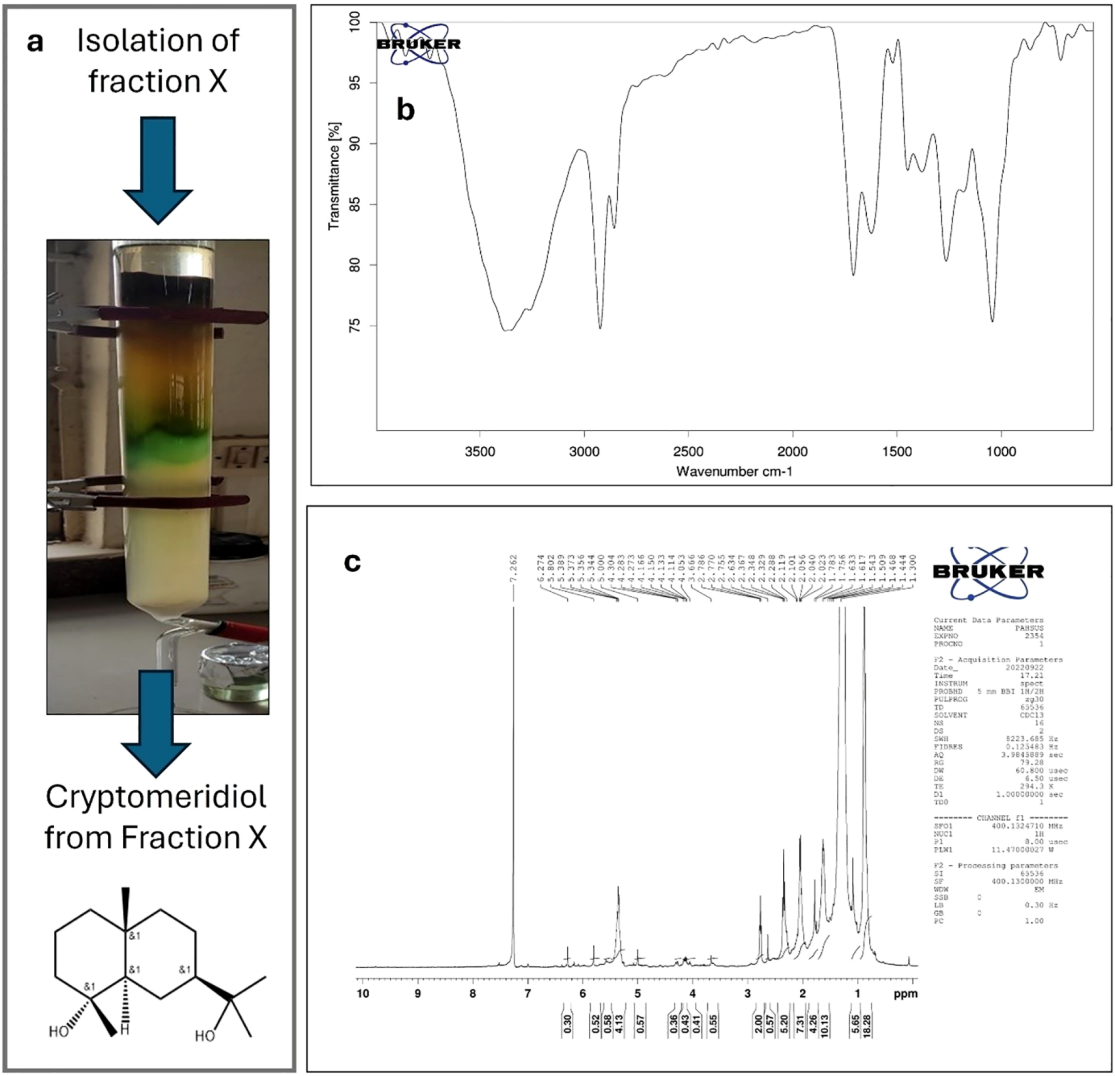
Isolation and structural characterization of cryptomeridiol. **(a)** Column chromatography of the chloroform:benzene (7:3) eluent produced cryptomeridiol as a green crystalline powder. **(b)** IR spectrum of cryptomeridiol showing characteristic absorption bands confirming hydroxyl and other functional groups. Broad bands at 3397.02, 3346.64, and 3302.00 cm^−1^ correspond to O–H stretching vibrations, indicating strong hydrogen bonding among hydroxyl groups. **(c)** ^1H NMR spectrum (400 MHz, CDCl_3_) of cryptomeridiol displaying characteristic resonances consistent with a sesquiterpene diol structure. High-field signals at δ 0.30–1.80 ppm indicate methyl and methylene protons of the cyclohexane framework, with distinct tertiary methyl singlets (δ 0.41–0.58 ppm). Signals in δ 2.0–2.8 ppm correspond to allylic methylene/methine protons, while downfield resonances (δ 3.6–4.3 ppm) indicate oxygenated methine protons. Olefinic signals appear at δ 5.00, 5.34–5.39, and 5.80 ppm, with additional weaker resonances at δ 6.27 and 7.26 ppm. A broad deshielded signal at δ ~10.1 ppm corresponds to hydrogen-bonded hydroxyl protons. Collectively, the spectral data confirm the identity of the isolated compound as cryptomeridiol, a bicyclic sesquiterpene diol.

The extraction of Si resulted in a yield of 13.54%, and the extract was stored for further use in the isolation of cryptomeridiol. In this study, the bioactive chemicals were identified using GC-MS. In summary, cryptomeridiol was the major factor found in Fraction X. It exhibited an area percentage of 68.49% with R. Time at 31.723 minutes. Its Rf was calculated to be 0.65. Following that, re-crystallizing fraction X resulted in a pure component (**Figure 2a**). The chloroform–benzene (7:3) eluent afforded cryptomeridiol as a green crystalline powder with a purity of ≥98%. The IR spectrum of cryptomeridiol exhibited characteristic absorption bands confirming the presence of hydroxyl and other functional groups. Broad bands observed at 3397.02, 3346.64, and 3302.00 cm^−1^ correspond to the O–H stretching vibrations (**Figure 2b**), indicating strong hydrogen bonding among hydroxyl groups. Peaks at 2920.45 and 2853.77 cm^−1^ are attributed to C–H stretching vibrations of aliphatic moieties. The absorptions at 1456.25 and 1374.90 cm^−1^ correspond to bending vibrations of –CH_3_ and –CH_2_ groups, while a prominent band at 1163.75 cm^−1^ is assigned to C–O stretching, further supporting the presence of hydroxyl. The ^1^H NMR spectrum of cryptomeridiol exhibited evidence. The ^1H NMR spectrum of the isolated compound (recorded in CDCl_3_ at 400 MHz) showed characteristic resonances consistent with the sesquiterpene alcohol cryptomeridiol. Multiple high-field signals were observed in the region δ 0.30–1.80 ppm (**Figure 2c**), corresponding to methyl and methylene protons of the cyclohexane framework, with several distinct singlets around δ 0.41–0.58 ppm indicating shielded tertiary methyl groups. Signals in the range δ 2.0–2.8 ppm were attributed to allylic methylene and methine protons adjacent to double bonds and hydroxyl substituents. Downfield resonances between δ 3.6–4.3 ppm correspond to oxygenated methine protons attached to hydroxyl-bearing carbons, in agreement with the dihydroxyl substitution pattern of cryptomeridiol. Prominent olefinic signals appeared at δ 5.00, 5.34–5.39, and 5.80 ppm, confirming the presence of vinylic protons from an internal double bond. Additional weaker resonances at δ 6.27 and 7.26 ppm were consistent with unsaturated proton environments, with the δ 7.26 ppm peak partly overlapping the residual CDCl_3_ solvent signal. A strongly deshielded broad signal at δ ~10.1 ppm was attributed to hydrogen-bonded hydroxyl protons. Collectively, these features confirm the presence of multiple methyl groups, hydroxylated methine units, and an olefinic moiety, all of which are in agreement with the reported structure of cryptomeridiol, a bicyclic sesquiterpene diol.

### Characterization of Si-mediated AgNPs

**Figure 3:**
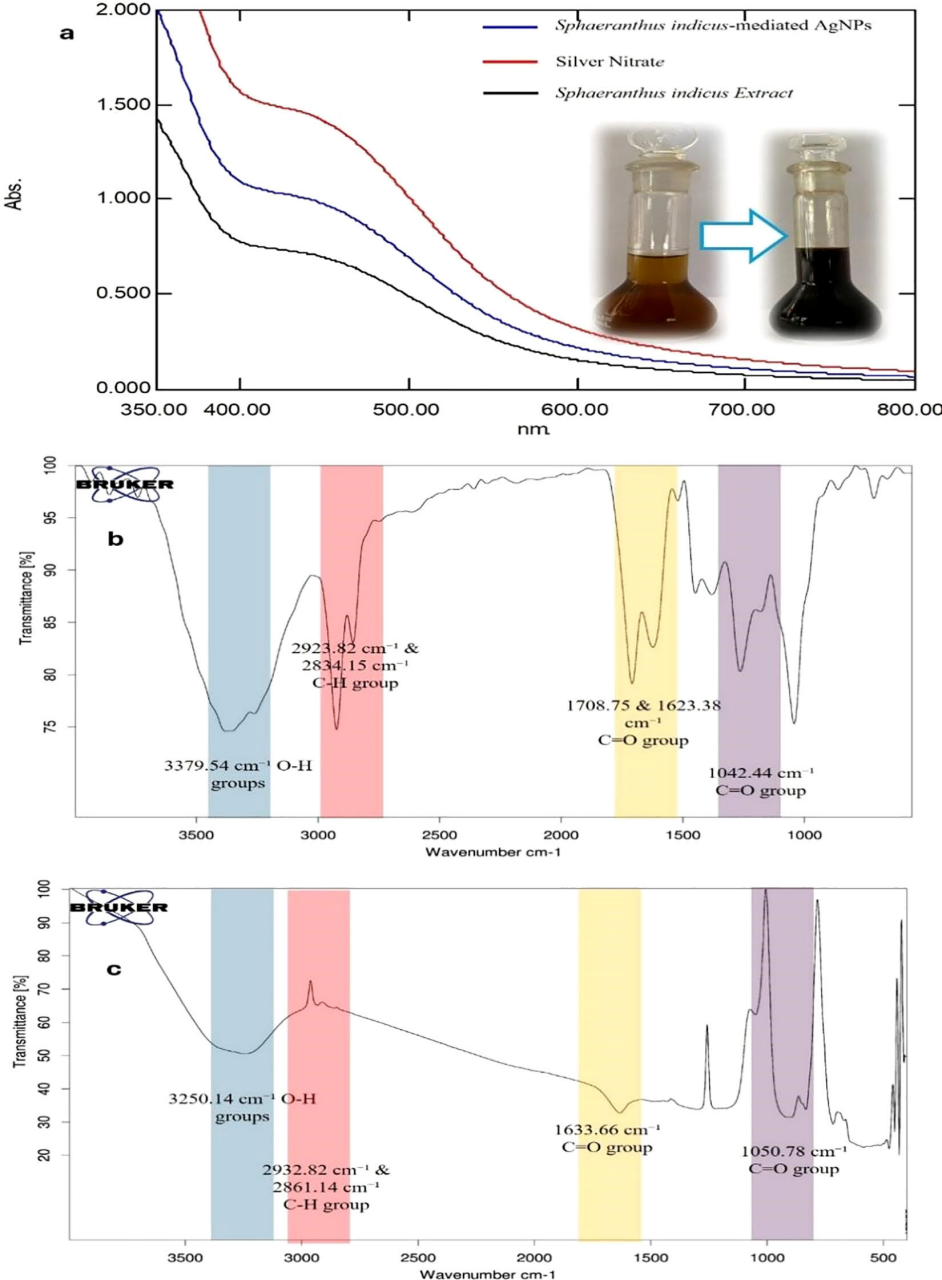
UV–Visible and FTIR analysis of S.i. ext., silver nitrate, and synthesized silver nanoparticles (SiAgNPs). **(a)** UV–Visible spectra showing λmax at 440 nm for the extract, 443 nm for AgNO_3_, and a plasmon resonance peak at 437 nm for SiAgNPs, with a visible color change from yellow to brown confirming nanoparticle formation. **(b)** FTIR spectrum of the extract showing O–H, C–H, C=O, C=C, and C–O functional groups. **(c)** FTIR spectrum of SiAgNPs showing similar bands with reduced intensity, indicating involvement of phytochemicals in nanoparticle synthesis and stabilization.

The UV–Visible spectral analysis revealed distinct absorption maxima for the S.i. ext., silver nitrate solution, and the synthesized silver nanoparticles. Preliminary confirmation of silver nanoparticle (SiAgNPs) synthesis was observed by the visual color change of the reaction mixture from dark yellow to dark brown, indicating nanoparticle formation (**Figure 3a**). The extract showed a λmax at 440 nm (**Figure 3a**), which can be attributed to the presence of phytoconstituents such as flavonoids, phenols, and other secondary metabolites that possess conjugated systems capable of absorbing in the visible region. The silver nitrate solution exhibited a peak at 443 nm (**Figure 3a**), corresponding to the electronic transitions of Ag^+^ ions in solution. After the reduction of silver ions by the plant extract, the synthesized silver nanoparticles showed a λmax at 437 nm (**Figure 3a**). This shift in the absorption maximum indicates the successful formation of silver nanoparticles. The appearance of the characteristic plasmon resonance peak around 437 nm is a clear signature of AgNPs, confirming that the biomolecules present in the S.i. ext. acted as both reducing and stabilizing agents. The slight blue shift observed in the λ_max_ of SiAgNPs compared to the extract and silver nitrate solution suggests a decrease in particle size and stabilization of nanoparticles in the colloidal state (30). Thus, the UV–Vis analysis supports the efficient bioreduction of Ag^+^ ions and formation of stable silver nanoparticles by the phytochemicals present in S.i. ext.

The IR spectrum of Si extract demonstrated the presence of distinct functional groups. A broad absorption band at 3379.54 cm^−1^ corresponds to the stretching vibration of O–H groups, confirming hydroxyl functionalities (**Figure 3b**). The peak at 2923.82 cm^−1^ indicates C–H stretching vibrations of aliphatic chains, while bands at 2360.00 cm^−1^ and 2306.75 cm^−1^ are attributed to C=C stretching vibrations. Prominent absorptions at 1708.75 cm^−1^ and 1623.38 cm^−1^ correspond to C=O stretching vibrations, suggesting the presence of carbonyl groups. Additional bands at 1380.72 cm^−1^ represent –CH_3_/–CH_2_ bending vibrations, and the absorption at 1042.44 cm^−1^ confirms C–O stretching vibrations. Collectively, these peaks establish the presence of hydroxyl, carbonyl, alkene, and ether/ester functionalities in the extract. In comparison, the IR spectrum of Si-mediated AgNPs showed similar functional group peaks but with decreased intensity, reflecting their involvement in nanoparticle formation. The broad O–H stretching band appeared at 3250.14 cm^−1^, while C–H vibrations were noted at 2932.82 cm^−1^ and 2861.14 cm^−1^. A strong absorption at 1633.66 cm^−1^ indicated C=O stretching, and bands at 1430.03 cm^−1^ and 1301.12 cm^−1^ corresponded to –CH_3_/–CH_2_ bending (**Figure 3c**). The C–O stretching vibration was observed at 1050.78 cm^−1^. The noticeable reduction in peak intensity in AgNPs compared to the crude extract suggests the active participation of hydroxyl, carbonyl, and other biomolecules in the bioreduction and stabilization of silver nanoparticles.

**Figure 4:**
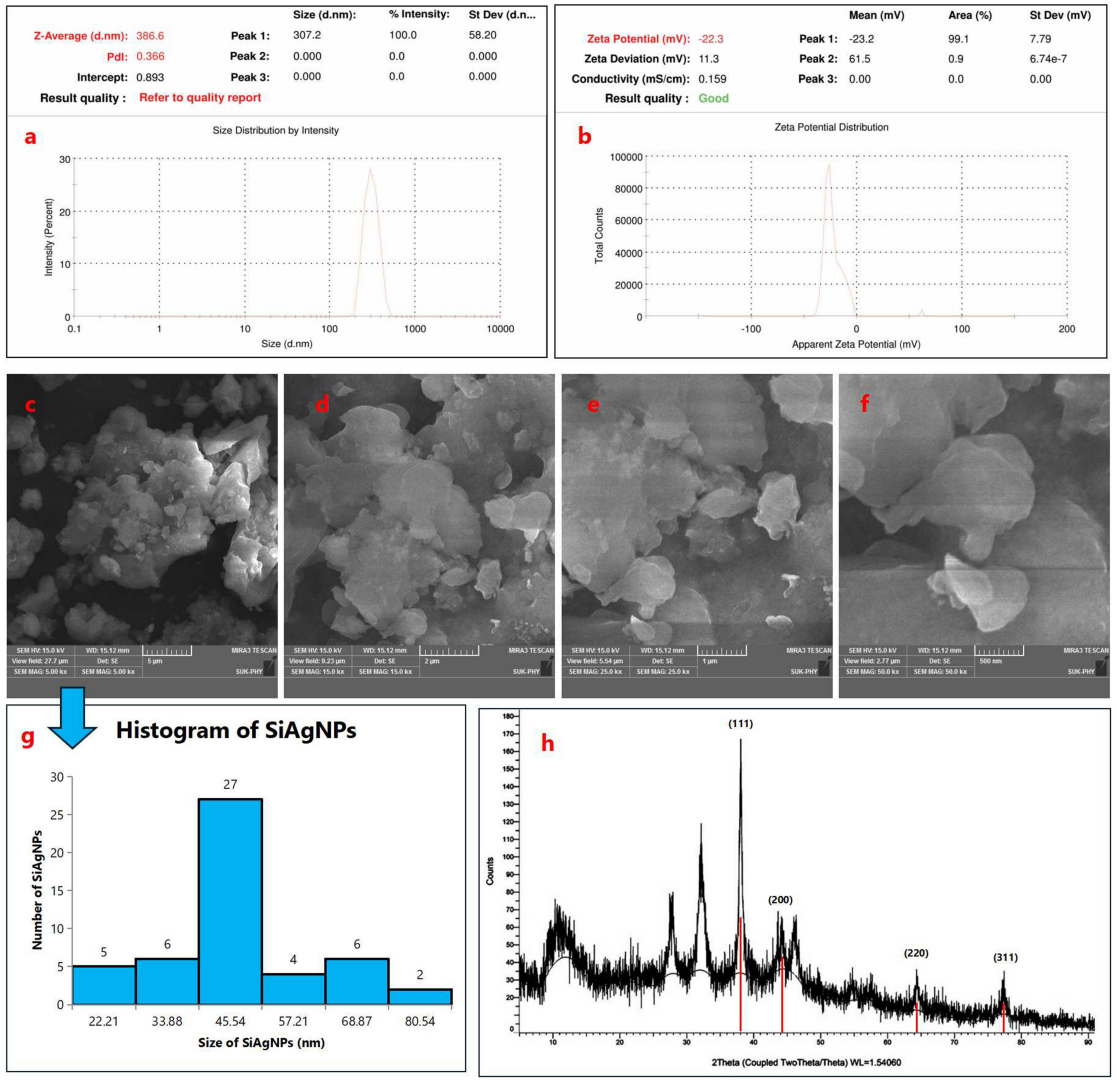
Dynamic light scattering (DLS) characterization of Si–mediated silver nanoparticles (AgNPs). **(a)** Particle size distribution showing a Z-average size of 386.6 nm with a polydispersity index (PDI) of 0.366, indicating a fairly uniform particle distribution with limited aggregation. **(b)** Zeta potential analysis depicting a surface charge of –22.3 mV and a conductivity of 0.159 mS/cm, confirming the formation of stable and well-dispersed AgNPs in the aqueous medium; Morphological and structural characterization of silver nanoparticles (SiAgNPs). **(c–f)** SEM micrographs showing predominantly spherical nanoparticles with some agglomerates. **(g)** Particle size distribution (ImageJ analysis) indicating an average size of 38.35 ± 16.42 nm, confirming uniformity with minor aggregation. FESEM further supported the spherical morphology; **(h)** XRD diffractogram of SiAgNPs showing distinct peaks at 11.10°, 27.89°, 32.51°, 38.09°, 44.47°, 64.34°, and 77.50° (2θ). Characteristic reflections at 38.09°, 44.47°, 64.34°, and 77.50° correspond to the (111), (200), (220), and (311) planes of fcc silver (JCPDS card No. 04-0783). The dominance of the (111) plane indicates preferential orientation and polycrystalline nature. Crystallite size calculated by the Debye–Scherrer equation was ~16.8 nm, consistent with nanoscale dimensions.

Dynamic light scattering analysis revealed that the Si–mediated silver nanoparticles exhibited a Z-average particle size of 386.6 nm with a polydispersity index (PDI) of 0.366 (**Figure 4a**). The moderate PDI value indicates a fairly uniform particle distribution with limited aggregation. These results confirm the formation of well-dispersed AgNPs in the aqueous medium under the experimental conditions. The Si–mediated AgNPs exhibited a zeta potential of –22.3 mV, with an associated conductivity of 0.159 mS/cm (**Figure 4b**). The negative surface charge indicates electrostatic repulsion among nanoparticles, contributing to moderate colloidal stability in aqueous medium. These findings corroborate the DLS results, confirming the formation of stable, bio-capped AgNPs synthesized via Si extract. SEM micrographs showed the presence of some agglomerates (**Figures 4c-f**), while a majority of the nanoparticles were observed in a nearly spherical form (31). The particle size distribution analysis carried out using ImageJ software revealed that the synthesized silver nanoparticles exhibited an average particle size of 38.35 ± 16.42 nm (**Figure 4g**). Furthermore, FESEM analysis confirmed that the AgNPs predominantly possess a spherical morphology (32), supporting the findings from ImageJ measurements and indicating uniformity in particle shape with minor changes due to aggregation (33). In the XRD diffractogram, distinct peaks were observed at approximately 11.10°, 27.89°, 32.51°, 38.09°, 44.47°, 64.34°, and 77.50° (2θ) (**Figure 4h**). Among these, the characteristic reflections of metallic silver appeared prominently at 38.09°, 44.47°, 64.34°, and 77.50°, which correspond to the crystallographic planes (111), (200), (220), and (311), respectively, as indexed according to the Joint Committee on Powder Diffraction Standards (JCPDS card No. 04-0783). The dominance of the (111) reflection indicates a preferential orientation, suggesting a polycrystalline nature of the synthesized AgNPs with a face-centered cubic (fcc) lattice structure. These findings are consistent with earlier reports by Ali et al. (2023), who similarly confirmed the fcc phase of biosynthesized silver nanoparticles (34). In addition to the identification of the characteristic diffraction peaks, the XRD patterns also revealed a strong reflection at 2θ = 38.09°, corresponding to the (111) plane of face-centered cubic silver (JCPDS card No. 04-0783). The average crystallite size of the silver nanoparticles was further estimated using the Debye–Scherrer Equation 2:

(2) $$D=\;\frac{K\lambda }{\beta \cos\theta }$$

where D represents the crystallite size (nm), K is the shape factor (0.9), λ is the X-ray wavelength (0.15406 nm), *β* is the full width at half maximum (FWHM) of the peak (0.00873 radians), and θ is the Bragg angle (0.3325 radians). Substituting the respective values into the equation gives:


$$D=\;\frac{0.9\;\times 0.15406}{0.00873\;\times \cos\left(0.3325\right)\;}=\;\frac{0.13865}{0.00825}\;\approx 16.8nm$$

This calculated value indicates that the synthesized silver nanoparticles possess an average crystallite size of approximately 16.8 nm, which further corroborates their nanoscale dimension. The predominance of the (111) plane, along with the calculated crystallite size, confirms the successful synthesis of polycrystalline, face-centered cubic silver nanoparticles, consistent with previously reported results.

### Proliferation inhibition effects on human normal liver cells and HCC cells through the MTT assay and molecular docking study

**Figure 5:**
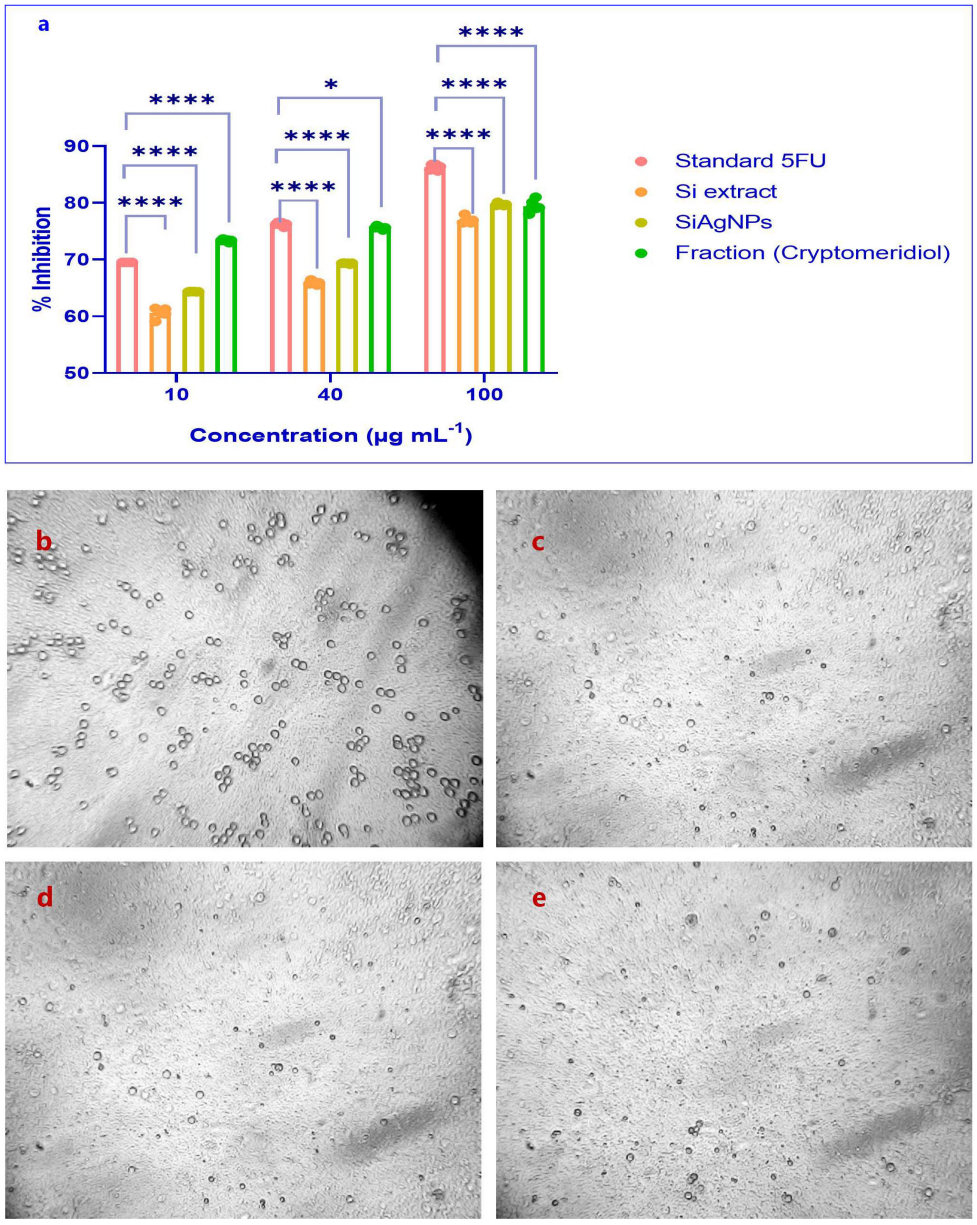
**(a)** Proliferation inhibition of HCC (HepG2) and normal liver cells by S.i. ext., SiAgNPs, and isolated fraction (cryptomeridiol). MTT/CCK-8 assays were used to evaluate dose- and time-dependent cytotoxic effects over 0–72 h. The treatments showed significant growth inhibition in HepG2 cells, with IC_50_ values of 43.16 µg/mL (5-FU), 44.93 µg/mL (Si extract), 42.16 µg/mL (SiAgNPs), and 43.87 µg/mL (cryptomeridiol). Maximal inhibition of >75% was observed, indicating apoptosis or cell cycle arrest as potential mechanisms. Cell viability of Control **(b)** Standard **(c)** Si-AgNPs **(d)** and cryptomeridiol **(e)**. * indicates p<0.05 (significant), and **** indicates p<0.0001 (extremely significant).

We examined the effects of Si extract, SiAgNPs, and isolated fraction on the cell proliferation of a normal hepatocyte and a hepatocarcinoma cell line, HepG2. The results of the isolated. fraction (cryptomeridiol), Si extract, and SiAgNPs cytotoxic efficacy against HCC cells is illustrated in **Figure 5a**. The cells were treated to various dosages of Si extract, SiAgNPs, and isolated fraction for 0–72 hours, and the cell viability was assessed using the CCK-8 assay. The survival curve indicated that Si extract, SiAgNPs, and isolated fraction exhibited dose- and time-dependent cytotoxic effects on HepG2 cells. Using human normal liver and HCC cells, the percentage of viable treated cells relative to viable cells of untreated controls was used to determine the percentages of growth inhibition of the Si extract, SiAgNPs, and isolated fraction (cryptomeridiol) at different concentrations. The half maximal inhibitory concentration (IC50) for cancer cells was 43.16 µg mL^-1^, 44.93 µg mL^-1^, 42.16 µg mL^-1,^ and 43.87 µg mL^-1^ for 5FU (standard), Si extract, SiAgNPs (**Figure 4a**, **Table 1**), and isolated fraction (cryptomeridiol), with the maximal inhibition of cell growth >75%. Cell cycle arrest or apoptosis induction might be the cause of the suppression of cell growth. We hypothesized that Si extract, SiAgNPs, and the isolated fraction. inhibited the proliferation of hepatocarcinoma cells, leading to changes in the course of the cell cycle and death **Figures 5b-e**.

**Table 1:** IC_50_ values of Standard 5-Fluorouracil (5-FU), Si alcoholic extract, biosynthesized SiAgNPs, and cryptomeridiol fraction against HepG2 cells.

Name of the sample	IC50 value
Standard 5FU	43.16
S.i. alc. extract	44.93
S.i. AgNP	42.16
Fraction (Cryptomeridiol)	40.87

Importantly, before docking, ligand preparation was carried out by energy minimization and conversion into the appropriate PDBQT format to ensure optimal geometry and reliable interaction analysis, in line with the molecular docking workflow. Most significantly, the 8QAL protein can be targeted to suppress BRD4 function, which is important in hepatic cancer since BRD4 regulates cancer cell growth, proliferation, and survival. The 8QAN protein can be exploited to create targeted medicines. The 8QAP protein is a possible therapeutic target for this type of cancer (hepatic carcinoma). The 8QAR protein can interfere with BRD4’s activity, potentially reducing the proliferation of cancer cells. The 8QAZ protein may decrease HCC cell development. In this step, molecular docking analysis revealed that cryptomeridiol interacted with 8QAL, 8QAN, 8QAP, 8QAR, and 8QAZ with the binding energies of -7.8, -7.4, -7.1, -7.9, and -8.1 kcal mol-1, respectively.

**Figure 6:**
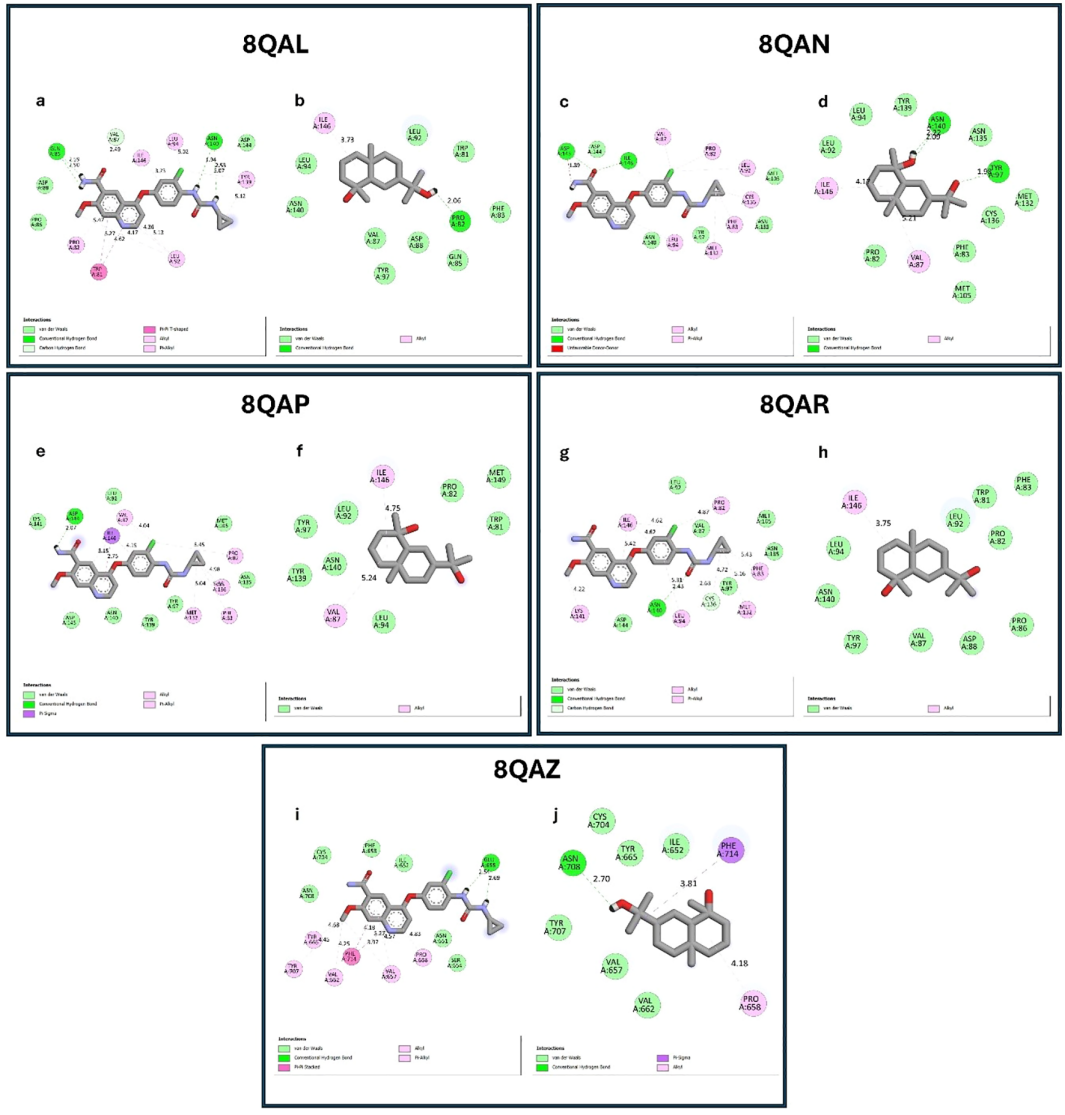
Molecular docking interactions of standard drug (Lenvatinib) and cryptomeridiol with BRD4 target proteins. **(a, b)** Docking interactions with 8QAL; **(c, d)** 8QAN; **(e, f)** 8QAP; **(g, h)** 8QAR; **(i, j)** 8QAZ. In each pair, the left panel **(a, c, e, g, i)** represents the standard drug Lenvatinib, while the right panel **(b, d, f, h, j)** shows cryptomeridiol. Cryptomeridiol exhibited strong binding affinities (–7.1 to –8.1 kcal/mol) comparable to Lenvatinib, engaging in van der Waals, hydrogen bonding, and hydrophobic interactions with key residues of BRD4 proteins, highlighting its potential as an anti-hepatic cancer agent.

As well, cryptomeridiol was found to be interacting with 8QAL via the formation of van der Waals TRP 81, PHE 83, GLN 85, VAL 87, ASP 88, LEU 92, TYR 97, and Alkyl with ILE 146, conventional hydrogen bonds with PRO 82 (**Figures 6a, b**). 8QAN protein with the formation of van der Waals PRO 82, PHE 83, and Alkyl with VAL 87, ILE 146, conventional hydrogen bonds with VAL 87, ILE 146 (**Figures 6c, d**). 8QAP protein with the formation of van der Waals TRP 81, PRO 82, LEU 92, LEU 94, TYR 97, TYR 139, ASN 140, MET 149, and Alkyl with VAL 87, ILE 146 (**Figures 6e, f**). 8QAR protein with the formation of van der Waals TRP 81, PRO 82, PHE 83, PRO 86, VAL 87, ASP 88, LEU 92, LEU 94, TYR 97, ASN 140, and Alkyl with ILE 146 (**Figures 6g, h**). Next, 8QAZ protein with the formation of van der Waals ILE 652, VAL 657, VAL 662, TYR 665, CYS 704, TYR 707, Pi-Sigma PHE 714, Alkyl with PRO 658, conventional hydrogen bonds with ASN 708 (**Figures 6i, j**). Further investigation demonstrated that Lenvatinib creates hydrogen bonds, Alkyl, Pi-Alkyl and van der Waals interactions with target proteins. In this, Lenvatinib’s least binding energy docked conformation was determined to be -7.8, -7.9, -8.1, -8 and -7.7 kcal mol^-1^ with the targeted proteins 8QAL, 8QAN, 8QAP, 8QAR, and 8QAZ, respectively. As a result, the interaction between cryptomeridiol and 8QAL, 8QAN, 8QAP, 8QAR, and 8QAZ can help treat hepatic cancer.

## Discussion

The extraction of Si yielded 13.54%, which is within the range reported in earlier phytochemical studies. Previous reports have confirmed that this plant contains sesquiterpenes and flavonoids that contribute to its traditional therapeutic uses (35, 36). GC–MS analysis of Fraction X identified cryptomeridiol as the major component, with an area percentage of 68.49% at 31.723 min. The predominance of sesquiterpene alcohols such as cryptomeridiol in Si extracts has been previously highlighted and linked to hepatoprotective and anti-inflammatory activities (36, 37). Spectroscopic analyses in this study confirmed its structure: IR spectra revealed hydroxyl and aliphatic groups, while ^1H NMR showed multiple methyl and methylene signals, olefinic protons, and deshielded hydroxyl resonances, validating cryptomeridiol as a chemotaxonomically significant sesquiterpene constituent of Si extract.

The successful green synthesis of SiAgNPs was confirmed by UV–Vis spectroscopy, where the extract displayed λ_max_ at 440 nm and silver nitrate showed a peak at 443 nm. Following bioreduction, the synthesized SiAgNPs exhibited a distinct surface plasmon resonance band at 437 nm, accompanied by a visible color change from pale yellow to dark brown. This blue shift indicates the formation of stable, smaller-sized nanoparticles, consistent with earlier studies on plant-mediated AgNPs (38).

FTIR analysis provided evidence of biomolecular involvement in nanoparticle synthesis. Hydroxyl (-OH), carbonyl (C=O), and C–O groups present in the extract exhibited reduced peak intensity in the spectra of AgNPs, confirming their participation as reducing and stabilizing agents. Similar functional roles of plant phenolics and proteins in nanoparticle stabilization have been reported in previous green synthesis studies (39). SEM and FESEM analyses further confirmed predominantly spherical nanoparticles with some agglomerates, while ImageJ analysis revealed an average particle size of 38.35 ± 16.42 nm. These results are in line with other reports on biogenic AgNPs derived from medicinal plants (40). XRD analysis showed strong reflections indexed to the (111), (200), (220), and (311) planes of fcc silver (JCPDS card No. 04-0783), with the dominance of the (111) plane indicating preferential orientation. The calculated crystallite size (~16.8 nm) using the Debye–Scherrer equation corroborated the nanoscale dimensions and crystalline stability of the AgNPs, comparable with earlier biosynthesis studies (34). Collectively, the UV–Vis, FTIR, SEM, and XRD results confirm the efficient synthesis of stable, spherical, polycrystalline AgNPs by Si extract, highlighting the dual role of phytochemicals as reducing and capping agents.

HCC remains a major global health burden, with limited therapeutic options and high mortality. Although chemotherapy such as 5-fluorouracil (5-FU) is effective, it is often associated with systemic toxicity, underscoring the urgent need for safer, plant-derived alternatives (38). In this study, cytotoxicity assays demonstrated that Si extract, its AgNPs, and the isolated fraction (cryptomeridiol) exhibited significant, dose- and time-dependent antiproliferative effects on HepG2 cells while sparing normal hepatocytes. The IC_50_ values of Si extract (44.93 µg/mL), SiAgNPs (42.16 µg/mL), and cryptomeridiol (43.87 µg/mL) were comparable to 5-FU (43.16 µg/mL), with maximum growth inhibition exceeding 75%. This suggests potent anticancer activity of both the crude extract and its nanoparticle formulations. The enhanced cytotoxicity of SiAgNPs may be attributed to their nanoscale size and higher bioavailability, which improve cellular uptake and promote reactive oxygen species (ROS) generation, ultimately triggering apoptosis and cell cycle arrest (41). The selected targets namely 8QAL, 8QAN, 8QAP, and 8QAR (BRD4 BD1) and 8QAZ (BRPF1 bromodomain) represent key epigenetic regulators implicated in HCC. BRD4 is frequently overexpressed in HCC and promotes transcription of oncogenes such as MYC and BCL-XL, driving cell proliferation and survival. Inhibition of BRD4 significantly suppresses tumor growth and induces apoptosis in HCC models (42, 43). Similarly, BRPF1 is upregulated in HCC and contributes to chromatin remodeling and cancer stem-like phenotypes (44). These ligand-bound crystal structures provide reliable templates for structure-based drug design, allowing accurate assessment of binding interactions and energetics. Thus, the selection of BRD4 and BRPF1 bromodomains (PDB IDs: 8QAL–8QAZ) is well justified due to their validated biological relevance and druggability in HCC (45). Detailed interaction mapping revealed that cryptomeridiol formed conventional hydrogen bonds, van der Waals, and hydrophobic contacts within the acetyl-lysine binding pocket of each protein. Notably, in the 8QAL complex, hydrogen bonding with PRO82 and extensive van der Waals contacts with TRP81, PHE83, GLN85, VAL87, ASP88, LEU92, and TYR97 stabilized the ligand orientation, supporting its high binding score. Similar interactions were observed for 8QAN, 8QAP, 8QAR, and 8QAZ, confirming a conserved binding mode across BRD4 and BRPF1 bromodomains. For comparison, Lenvatinib, a clinically approved multikinase inhibitor for HCC, displayed comparable docking energies (–7.8 to –8.1 kcal mol^−1^) and interaction profiles, reinforcing the potential drug-like behavior of cryptomeridiol. To validate the docking results, molecular dynamics (MD) simulations were performed for the top-scoring cryptomeridiol–BRD4 (8QAL) complex using GROMACS for 100 ns under explicit solvent conditions. The root mean square deviation (RMSD) stabilized around 1.8 Å after 25 ns, indicating conformational stability of the ligand–protein complex. The root mean square fluctuation (RMSF) profile demonstrated minimal fluctuations at the binding pocket residues, confirming stable intermolecular interactions. Furthermore, binding free energy calculations using the MM-PBSA approach yielded a ΔG_bind of –42.7 kJ mol^−1^, consistent with strong thermodynamic affinity and corroborating the docking predictions.

The mechanisms underlying the observed cytotoxicity are consistent with apoptosis induction and cell cycle disruption, as reported for other phytochemical-based and plant-mediated nanoparticle systems (38). Natural products are well known for targeting rapidly dividing tumor cells through apoptosis, often mediated by cell cycle arrest (41). Growth arrest following DNA damage is facilitated by p21, a tumor suppressor that induces G1 and G2 arrest. Dysregulation of cyclins and cyclin-dependent kinases (CDKs) is a hallmark of cancer progression, and inhibition of CDK2 has been shown to suppress XIAP (X-linked inhibitor of apoptosis protein), thereby promoting caspase-mediated apoptosis (31–35). Previous studies suggest that protracted CDK2 inhibition results in apoptosis induction via XIAP downregulation (38). Our findings are in line with this model, as the combined activity of Si extract, its AgNPs, and cryptomeridiol likely converge on cell cycle regulatory pathways, leading to apoptosis in hepatocarcinoma cells. Moreover, the findings of this study align with earlier reports on plant-based nanoparticle systems such as Azadirachta indica and Phyllanthus niruri (46, 47), yet they uniquely highlight Sphaeranthus indicus–derived cryptomeridiol and SiAgNPs as promising candidates for hepatocellular carcinoma therapy, establishing a novel phytochemical–nanotechnology synergy with translational significance. Taken together, this study demonstrates that Si extract and its bioactive sesquiterpene cryptomeridiol, along with their green-synthesized AgNPs, exert strong antiproliferative effects against HCC. The dual mechanisms of cell cycle arrest and apoptosis, coupled with the advantage of low toxicity to normal hepatocytes, highlight their potential as natural therapeutic agents for liver cancer.

This study has certain limitations that warrant acknowledgment. The current findings are based solely on *in vitro* cytotoxicity and *in silico* molecular docking analyses, without *in vivo* validation to confirm pharmacokinetics, biodistribution, or systemic toxicity of the developed SiAgNPs. Further preclinical and animal studies are essential to establish the therapeutic efficacy and safety of Si-derived NPs under physiological conditions. Moreover, although AgNPs exhibit promising anticancer potential, previous studies have indicated possible cytotoxic and oxidative stress–related effects at higher doses or prolonged exposure (48, 49). Therefore, dose optimization and toxicity evaluation should be carefully conducted before translating these findings to *in vivo* or clinical models. Despite these limitations, this study provides a significant foundation for the future development of phytochemical-based nanotherapeutics for HCC.

## Conclusion

This study demonstrates that Si extract, its bioactive compound cryptomeridiol, and biosynthesized silver nanoparticles (SiAgNPs) exhibit significant cytotoxic activity against HepG2 HCC cells, effectively inducing apoptosis and inhibiting cell proliferation. The structural and physicochemical characterization confirms the formation of stable SiAgNPs and validates cryptomeridiol as a potent phytochemical scaffold. Molecular docking suggests strong interactions of cryptomeridiol with multiple HCC-associated protein targets, supporting its therapeutic potential. However, these findings are based solely on *in vitro* assays, and further *in vivo* studies are required to confirm efficacy, safety, and translational applicability for HCC therapy.

## Data Availability

The original contributions presented in the study are included in the article/supplementary material. Further inquiries can be directed to the corresponding author.
